# Inflammatory Semaphorins in the Pathogenesis and Prognosis of Acute Ischemic Stroke

**DOI:** 10.3390/medicina61112060

**Published:** 2025-11-19

**Authors:** Esen Çiçekli, Dilcan Kotan, Levent Avcı

**Affiliations:** 1 Sakarya University Training and Research Hospital, 54400 Sakarya, Türkiye; esencirit@gmail.com (E.Ç.); leventavcii07@gmail.com (L.A.); 2 Department of Neurology, Sakarya University Faculty of Medicine, 54100 Sakarya, Türkiye

**Keywords:** ischemic stroke, semaphorin 3A, semaphorin 3F, semaphorin 4A, semaphorin 4D, semaphorin 7A

## Abstract

*Background and Objectives*: Semaphorins are immunoregulatory proteins involved in inflammation and neurovascular modulation. Their roles in ischemic stroke pathogenesis and prognosis have recently gained attention. This study aimed to evaluate serum levels of semaphorin 3A, 3F, 4A, 4D, and 7A in patients with acute ischemic stroke and investigate their relationship with disease severity and prognosis. *Materials and Methods*: A total of 45 patients with acute ischemic stroke and 39 control individuals were enrolled. Serum semaphorin levels were measured using ELISA. Clinical data, including TOAST classification, NIHSS scores, and laboratory parameters, were recorded. Correlations between semaphorin levels and clinical or biochemical variables were analyzed statistically. *Results*: Semaphorin 4A levels were significantly lower and semaphorin 7A levels significantly higher in the patient group compared to controls (*p* < 0.001). Semaphorin 7A positively correlated with NIHSS scores (r = 0.390. *p* = 0.008). Semaphorin 3A and 4A levels showed significant correlations with inflammatory markers and lipid profiles. Semaphorin 3A was higher in female patients. No associations were found with TOAST subtypes or treatment modalities. Five (11.1%) patients died due to stroke-related complications, no significant differences in semaphorin levels were observed between survivors and non-survivors. *Conclusions*: Semaphorin 3A, 4A, and 7A levels may serve as potential biomarkers for inflammation and disease severity in acute ischemic stroke. Semaphorin 7A, in particular, showed strong prognostic value due to its association with stroke severity. These findings suggest that semaphorins could aid in clinical risk stratification and early intervention planning in ischemic stroke.

## 1. Introduction

Acute ischemic stroke is a sudden-onset neurological emergency caused by disruption or significant reduction in blood flow to the brain, often resulting in permanent neurological deficits or death [1]. This disruption leads to a deficiency of oxygen, glucose, and lipids, subsequently reducing adenosine triphosphate (ATP) production. Lactic acidosis develops, disrupting cellular homeostasis and leading to necrosis of the brain parenchyma [2]. In addition to excitotoxicity, mechanisms like oxidative stress, inflammation, and apoptosis play critical roles in ischemic stroke. Particularly, oxidative stress, excitotoxicity, and Damage-associated Molecular Patterns (DAMPs) released by dead cells activate microglia. This activation triggers the release of proinflammatory mediators, potentially worsening brain injury [3,4]. Numerous studies have shown that inflammatory mechanisms play very complex roles in the progression and pathogenesis of ischemic stroke [5]. Semaphorins have been shown in a number of studies to affect the prognosis of ischemic stroke by regulating the neurovascular unit. Although semaphorins were first recognized as guide molecules in neuronal development, they are recognized as extracellular signaling proteins found in many systems such as musculoskeletal, cardiovascular, respiratory, and immune systems [6]. Semaphorins have more than 20 members. They exert their effects by binding to the neuropilin (Npn) and plexin (Plxn) protein families, which are important receptors [7]. Semaphorin 3A is a glycoprotein secreted from many human cell types. It plays different pathogenetic roles in human body including the cardiovascular system diseases, angiogenesis, tumor metastasis, and the immune system [8]. It has been found that semaphorin 3A is closely related to ischemic stroke and is effective in the recovery of stroke [9]. Semaphorin 3F, a potent angiogenesis inhibitor, exerts its effect on Npn-1 during angiogenesis and competes with vascular endothelial growth factor (VEGF), which is a potent pro-angiogenic cytokine [10]. Semaphorin 4A plays an important role in the regulation of T cell hemostasis, activation, and differentiation of Th1, Th2, and Th17 [11]. Semaphorin 4D, also known as CD100, is an immune semaphorin expressed by T lymphocytes, eosinophils, dendritic cells (DC) and B lymphocytes. In addition, overexpression of semaphorin 4D has been observed in tumors such as osteosarcoma, colon cancer, and lung cancer [12]. Semaphorin 7A is an immune semaphorin involved in various immunological and inflammatory processes. It also plays a role in the proliferation and angiogenesis of tumor cells and is closely related to tumor pathogenesis. In this study, we aimed to investigate the relationship between the Semaphorin 3A, 3F, 4A, 4D and 7A which are immunoregulatory semaphorins have been known to provide axon growth for more than thirty years, with acute ischemic stroke which has various immunological and inflammatory mechanisms in its pathophysiology.

## 2. Materials and Methods

### 2.1. Study Population

All patients who were admitted to the Emergency Department of Sakarya University Faculty of Medicine Training and Research Hospital, diagnosed with acute ischemic stroke according to WHO criteria and hospitalized, were included in our study. The patients were informed about the study by the physician, and a written consent form was obtained. During the first admission to the emergency department, vital signs, systemic and neurological examination findings of the patients were recorded. The form prepared for patients diagnosed with acute ischemic stroke was filled out. In this form, the patient’s identity information, admission complaints, medications and medical history (DM, HT, ischemic heart disease, carotid stenosis, hyperlipidemia, malignancy, smoking and alcohol use) were recorded. Computed tomography (CT) and magnetic resonance imaging (MRI) examinations taken in our emergency department determined the localization of the infarction and recorded in writing by the radiologist. Hemoglobin, hematocrit (Hct), platelet, glucose, urea and creatinine levels were recorded. Finally, the duration and outcomes of treatment in the clinic where the patients were hospitalized were recorded.

Serum samples to be obtained from routine blood tests of the patients in the emergency department were stored at −80 °C until the study date.

The control group consisted of age- and sex-matched volunteers without any neurological or autoimmune diseases. Common vascular risk factors such as hypertension, diabetes mellitus, and smoking were not exclusion criteria, in order to better reflect the general population. After written consent was obtained from the control individuals, demographic and clinical information was accessed and recorded. Serum samples obtained from blood samples were stored at −80 °C until the study date.

#### 2.1.1. Inclusion Criteria for Patients

- Patients over the age of 45 who were admitted to the emergency department within the first 24 h after acute stroke.

#### 2.1.2. Inclusion Criteria for the Control Group

- No previous history of stroke.

#### 2.1.3. Exclusion Criteria for Patients

- Not admitted to the hospital within the first 24 h of ischemic stroke;
- Inability to reach the patient’s blood sample;
- Inability to access anamnesis from the file or incomplete anamnesis;
- The patient’s desire to withdraw from the study.

#### 2.1.4. Exclusion Criteria for the Control Group

- Previous stroke history.

### 2.2. Evaluation Criteria

In the study, it was evaluated whether there was a difference between the patient and the control group in terms of serum semaphorin 3A, 3F, 4A, 4D, 7A levels. In the patient group, semaphorin levels were compared according to the risk factors, age and gender characteristics of the patients. TOAST (Trial of ORG 10172 in Acute Stroke Treatment) classification and NIHSS (National Institutes of Health Stroke Scale) score and the relationship between semaphorin levels were examined for determining the stroke risk and clinical severity. Clinical outcomes, including stroke severity (NIHSS score) and mortality, were recorded to evaluate prognosis.

None of the patients received intravenous thrombolysis or endovascular reperfusion therapy prior to blood sampling. All samples were obtained within the first 24 h after stroke onset.

### 2.3. Study of Semaphorin Levels in Serum Samples by ELISA Method in the Laboratory

Semaphorin levels in serum samples in the patient and control groups were studied by specialized personnel in the laboratory where the kits were supplied. In this process, appropriate ELISA kits (MyBioSource, San Diego, CA, USA) were used to quantitatively measure human semaphorin concentrations in serum, plasma, and other biological fluids in vitro.

5–10 mL of blood was taken from the patients in the BD Vacutainer SST II Advance tube and kept at room temperature for 10–20 min. It was centrifuged at 2000–3000 RPM for 20 min and the supernatant part was removed and placed in Eppendorf tubes until the operating time and stored at −80 °C as a backup. Likewise, Semaphorin 3A, 3F, 4A, 4D, 7A kits were stored in the storage conditions recommended by the company until the working day. Samples were brought to room temperature before the test began. Hemolyzed specimens were included in the study. To determine semaphorin levels in the study, ELISA kits were used for each human semaphorin subgroup (separately for Semaphorin 3A, 3F, 4A, 4D, 7A) in accordance with company recommendations. For this purpose, all reagents to be used in the study were brought to room temperature before the study. The standards in the kit were diluted with standard diluent and serial dilutions were made as recommended by the company according to the type of semaphorin to be studied. To detect semaphorin 3A, 3F, 4A, 4D, 7A levels were studied in Triturus Fully Automated ELISA Analyzer (Triturus, Grifols, Italy). The Triturus Fully Automated ELISA Analyzer is a 4-plate fully open and fully automated ELISA analyser used to test and process tests such as infectious diseases, autoimmunity, and biologic drug monitoring. It could read between 0–3000 OD with a spectral range of 405–620 nm. The device measures with the spectrophotometric measurement principle.

The working steps recommended by the company for semaphorin 3A, 3F, 4A, 4D, 7A (addition of standards, incubation, washing, conjugate addition, stopping stages, etc.) were defined in the device. After all the preliminary preparation processes were completed, the samples were numbered and loaded into the device carousel in the appropriate order, and the study was started. The study used micro-ELISA plates pre-coated with an antibody specific to each human semaphorin subgroup (separately for Semaphorin 3A, 3F, 4A, 4D, 7A).

Although there are differences according to the type of semaphore in the study stages, the common principle of the tests is: Samples were added to the microplate wells in the amount recommended by the company. Then, biotin-labeled detection antibody and conjugate were added and incubated, respectively. After the necessary incubation and washing stages, the reaction was terminated. All process steps were performed with the Triturus Fully Automatic ELISA Analyzer (Grifols, Barcelona, Spain).

### 2.4. Evaluation of Results

After the reaction was terminated with the stop solution, the colour change was measured spectrophotometrically in the same device at a wavelength of 450 nm ± 10 nm. For semaphorin 3A, 3F, 4A, 4D, 7A measurements, a standard curve was created by placing the absorbance (optical density and semaphorin concentration) on the x and y axis according to the semaphorin type. Then, regression analysis was performed.

### 2.5. Statistical Analyses

Statistical analyses were performed using IBM SPSS Statistics software (Version 21.0, IBM Corp., Armonk, NY, USA). The conformity of the variables to the normal distribution was examined by visual (histogram and probability graphs) and analytical methods (Shapiro–Wilk). Descriptive statistics were given using mean and standard deviation for normally distributed variables, median and interquartile intervals for normally non-distributed variables (frequency tables for categorical variables). The frequencies of categorical variables according to the desired groups were shown with cross-tabulations. Whether there was a difference in these frequencies between the groups was given using the Pearson Chi-Square Test. Mann–Whitney U test was used for group comparison of variables that are not normally distributed. One Way Anova Test was used for the comparison of more than two groups with normal distribution, and Kruskal–Wallis Test was used for the comparison of more than 2 groups that were not normally distributed. The Spearman Correlation Test was used to look at the relationship of variables that are not normally distributed. Cases where the *p* value was below 0.05 were considered statistically significant.

## 3. Results

### 3.1. Characteristics of Patients

There were 45 patients in the study group; 28 were male (62.2%) and 17 were female (37.8%). There were 21 males (54%) and 18 females (46%) in the control group. There was no significant difference between the groups in terms of gender characteristics (*p* = 0.437). The age of the patient group ranged from 36 to 86 years, with a mean of 65.22 ± 11.17. The control group ranged from 51 to 86 years, with a mean of 64.56 ± 9.50. The mean ages of the two groups did not differ significantly (*p* = 0.673) (Table 1).

Comorbidities included hypertension in 23 patients (51.1%), coronary artery disease in 13 (28.9%), diabetes mellitus in 12 (26.7%), cerebrovascular disease in 8 (17.8%), atrial fibrillation in 3 (6.7%), chronic renal failure in 3 (6.7%), congestive heart failure in 1 (2.2%), and hyperlipidemia in 1 (2.2%). A history of smoking was present in 68.9% of patients (Table 1). 

21 of stroke patients used antiplatelet agents (46.7%), 17 used antihypertensive therapy (37.8%), 12 used hypoglycemic therapy (26.7%), 4 used statin therapy (8.9%) and no patients used anticoagulants.

During hospitalization, 5 (11.1%) patients died due to stroke-related complications, while 40 (88.9%) survived. No deaths occurred in the control group.

### 3.2. Stroke Characteristics and TOAST Classification of Patients

Brain computed tomography (CT) and diffusion magnetic resonance imaging (MRI) examinations performed in the emergency department revealed acute ischemic stroke findings at the cortical level in 19 cases (42.2%), near the basal ganglia or lateral ventricles in 10 cases (22.2%), at the thalamus in 8 cases (17.7%), at multiple localizations in 7 cases (15.6%), and at the cerebellum in 1 case (2.2%). According to the TOAST classification, 10 patients were classified as large vessel occlusion (22.2%), 10 as small vessel disease (22.2%), 8 as cardioembolic (17.8%), and 17 as undetermined etiology (37.8%).

### 3.3. Comparison of Serum Semaphorin Levels Among Groups and Analysis of Their Associations with Clinical and Laboratory Variables

It was observed that semaphorin 4A levels were lower in the patient group than in the control group. and semaphorin 7A levels were higher in the patient group than in the control group (*p* < 0.001. *p* < 0.001) (Table 2).

In the patient group, women’s semaphorin 3A levels were higher than men’s (*p* = 0.036). There was no significant difference between gender in terms of semaphorin 3F, 4A, 4D, 7A levels in the patient group (*p* = 0.535. *p* = 0.128. *p* = 0.760. *p* = 0.981).

According to the data obtained, it was determined that there was a significant relationship between some semaphorin levels and routine blood parameters. A positive correlation was observed between ALT (alanine aminotransferase) and semaphorin 4A level (r = 0.435, *p* = 0.003). There is a positive correlation between albumin and semaphorin 3A levels (r = 0.534, *p* < 0.001). There is a negative correlation between albumin and semaphorin 7A levels (r = −0.412, *p* = 0.005). Positive correlations were found between total cholesterol (TC) and semaphorin 3A levels (r = 0.441, *p* = 0.004) and semaphorin 4A levels (r = 0.329, *p* = 0.029). There are positive correlations between LDL (Low Density Lipoprotein) and semaphorin 3A levels (r = 0.429, *p* = 0.006) and semaphorin 4A levels (r = 0.305, *p* = 0.044). A positive correlation was found between neutrophil (NEU) and semaphorin 4A levels (r = 0.330, *p* = 0.027). Positive correlations were found between hemoglobin (HGB) and semaphorin 4A levels (r = 0.349, *p* = 0.020). There are positive correlations between platelet (PLT) and semaphorin 3A levels (r = 0.346, *p* = 0.029). It was found that there was a positive correlation between monocyte (MNT) and semaphorin 4A level (r = 0.343, *p* = 0.023) (Table 3).

No significant differences were found between the medical treatments administered and semaphorin levels, including 3A (*p* = 0.413), 3F (*p* = 0.540), 4A (*p* = 0.794), 4D (*p* = 0.232), and 7A (*p* = 0.578).

It was determined that serum semaphorin 7A level increased as the NIHSS stroke score of the patient group increased (R = 0.390. *p* = 0.008).

There was no statistically significant difference between the TOAST class groups and the semaphorin 3A, 3F, 4A, 4D, 7A levels of the patients. (*p* > 0.05).

There was no significant difference in semaphorin 3A (*p* = 0.932), 3F (*p* = 0.598), 4A (*p* = 0.216), 4D (*p* = 0.484), or 7A (*p* = 0.447) levels between smoking and non-smoking patients.

Five (11.1%) patients died during hospitalization; no significant differences in semaphorin levels were found between survivors and non-survivors (*p* > 0.05)

## 4. Discussion

In our study, it was determined that semaphorin 7A levels were higher in the patient group than in the control group and there was a statistically significant relationship between it and the NIHSS stroke score. In support of our findings, another study reported that semaphorin 7A levels were observed high in patients with acute ischemic stroke due to large artery atherosclerosis, that it may play a role in atherosclerosis, and that it can be used in early diagnosis and risk assessment [13]. The fact that semaphorin 7A tends to increase as the NIHSS stroke score increases may be indicative of the inflammatory response, which intensifies as the severity of stroke increases, and this finding draws attention to the prognostic importance of semaphorin 7A in stroke. Similarly, there are studies in the literature on the clinical importance of semaphorin 7A in inflammatory diseases [14,15]. Albumin is a negative acute phase reactant synthesized in the liver and provides valuable information about the inflammatory and nutritional status of the body [16,17]. The negative correlational relationship between Semaphorin 7A and albumin levels in the patient group is also valuable in terms of indicating the inflammatory response in acute ischemic stroke. In many studies, it has been reported that there is a significant correlation between hypoalbuminemia and stroke severity in patients with acute ischemic stroke [18,19,20]. Semaphorin 7A appears to be a promising potential to control this intense inflammatory response, which is associated with neuronal toxicity and poor prognosis.

In line with previous reports indicating a higher prevalence of autoimmune and inflammatory diseases in women, Sema3A levels were higher in female patients [21,22]. However, this observation should be interpreted with caution. None of the participants had a diagnosed autoimmune disease, and the difference may reflect sex-related immunological or hormonal variation rather than autoimmune comorbidity.

When the relationship between hyperlipidemia, a known risk factor for acute ischemic stroke, and semaphorin levels was examined, we found that semaphorin 3A and 4A levels were positively correlated with total cholesterol and LDL levels. In a previous study investigating the role of Semaphorin 3A in lipid metabolism—which plays a crucial role in atherosclerosis development—it was reported that reduced expression of Semaphorin 3A has been shown to promote atherosclerotic plaque formation by increasing lipid accumulation and inflammation [23]. However, the positive correlation between semaphorin levels and lipid parameters, although statistically significant, should be interpreted with caution due to the small number of patients with overt hyperlipidemia. In ischemic stroke, blood–brain barrier (BBB) disruption plays a pivotal role in secondary neuronal injury. Semaphorins, particularly Sema3A and Sema4D, are known to influence endothelial permeability, tight junction integrity, and leukocyte migration across the BBB. Elevated levels of these proteins may therefore reflect enhanced BBB dysfunction and neuroinflammatory processes [24,25].

Higher hemoglobin and platelet counts observed in stroke patients may indicate a pro-inflammatory or hypercoagulable state that could also contribute to increased semaphorin expression, as previously suggested in studies linking hematologic parameters to inflammatory and vascular responses after stroke [26,27,28].

Semaphorins play important regulatory roles in the immune system and interact with immune cells [29]. They contribute to inflammatory processes through their interactions with different immune cells [30,31]. The significant association of semaphorin 4A with the levels of neutrophils and monocytes, which are inflammatory cells, suggests that semaphorin 4A has a critical role in directing the immune response and may be a potential target in regulating inflammation.

This study has several limitations. Functional outcome scales such as the modified Rankin Scale or Barthel Index were not included, as our primary focus was on biochemical markers rather than clinical scoring. Prognosis was evaluated indirectly through the NIHSS score and mortality data. Additionally, volumetric neuroimaging data were not available for all patients, which may have limited detailed assessment of infarct size. The relatively small sample size also restricts the generalizability of our findings. Future studies combining clinical, radiological, and biochemical parameters are warranted to validate these results.

## 5. Conclusions

In this study, we investigated the relationship between semaphorin 3A, 3F, 4A, 4D, and 7A levels and disease severity in patients with acute ischemic stroke. Semaphorin 3A and 4A levels were significantly associated with inflammatory and prothrombotic blood parameters, while semaphorin 7A levels correlated with the NIHSS stroke score, suggesting potential prognostic value. Only a limited number of studies have explored immune semaphorins in ischemic stroke, and our findings contribute to this growing field of research. Although semaphorins are recognized as potent immunoregulatory molecules, their diagnostic and prognostic roles in stroke remain underexplored. Further large-scale studies are warranted to validate these results.

## Figures and Tables

**Table 1:** Demographic and clinical characteristics of stroke patients and control subjects.

Parameter	Stroke Patients (*n* = 45)	Controls (*n* = 40)	* *p*-Value
Age (years, mean ± SD)	65.22 ± 11.17	64.56 ± 9.5	0.673
Sex (F/M)	17/28	18/21	0.437
Smoking, *n* (%)	31 (68.9)	11 (27.5)	0.001
Hypertension, *n* (%)	23 (51.1)	10 (25.0)	0.021
Diabetes mellitus, *n* (%)	12 (26.7)	5 (12.5)	0.090
Hyperlipidemia, *n* (%)	1 (2.2)	1 (2.5)	0.940
Coronary heart disease, *n* (%)	13 (28.9)	4 (10.0)	0.040
Atrial fibrillation, *n* (%)	3 (6.7)	1 (2.5)	0.310
Chronic kidney disease, *n* (%)	3 (6.7)	1 (2.5)	0.310

* Data are presented as mean ± standard deviation or *n* (%). *p* < 0.05 was considered statistically significant.

**Table 2:** Comparison of Semaphorin 3A, 3F, 4A, 4D, 7A levels of the patient and control group.

Semaphorin	Patient (Mean ± SD)	Control (Avg ± SS)	*p*-Value
3A	1.71 ± 2.11	2.37 ± 2.35	0.704
3F	9.08 ± 3.67	9.83 ± 3.46	0.087
4A	8.35 ± 3.29	14.86 ± 3.16	<0.001
4D	2.02 ± 1.34	3.01 ± 4.16	0.300
7A	3.33 ± 0.56	1.97 ± 0.52	<0.001

**Table 3:** The relationship between semaphorin levels and certain blood parameters in the patient group.

Parameter		3A	3F	4A	4D	7A
ALT	r	0.192	0.019	0.435	0.058	−0.109
	*p*	0.208	0.904	0.003	0.705	0.474
	*n*	45	45	45	45	45
Albumin	r	0.534	−0.051	0.046	−0.185	−0.412
	*p*	0.001	0.740	0.762	0.224	0.005
	*n*	45	45	45	45	45
Total Cholesterol	r	0.441	0.246	0.329	−0.093	−0.056
	*p*	0.004	0.104	0.029	0.543	0.714
	*n*	45	45	45	45	45
LDL	r	0.429	0.258	0.305	−0.049	0.069
	*p*	0.006	0.087	0.044	0.747	0.651
	*n*	45	45	45	45	45
Neutrophyl	r	0.017	0.038	0.330	−0.016	0.078
	*p*	0.912	0.802	0.027	0.917	0.610
	*n*	45	45	45	45	45
Hemoglobin	r	−0.123	0.145	0.349	0.168	0.124
	*p*	0.420	0.340	0.020	0.270	0.416
	*n*	45	45	45	45	45
Platelet	r	0.346	0.192	0.077	−0.134	−0.241
	*p*	0.029	0.207	0.614	0.381	0.110
	*n*	45	45	45	45	45
Monosyt	r	0.027	0.052	0.343	−0.153	−0.121
	*p*	0.861	0.736	0.023	0.314	0.428
	*n*	45	45	45	45	45
Lenfosyt	r	0.025	0.089	0.283	−0.026	−0.249
	*p*	0.873	0.561	0.060	0.866	0.099
	*n*	45	45	45	45	45

Note: The Spearman correlation test was used, and a *p*-value < 0.05 was considered statistically significant.

## Data Availability

The datasets generated and/or analyzed during the current study are available from the corresponding author on reasonable request.
